# Factors associated with residual urine volume preservation in patients undergoing hemodialysis for end-stage kidney disease in Kinshasa

**DOI:** 10.1186/s12882-018-0865-x

**Published:** 2018-03-20

**Authors:** Vieux Momeme Mokoli, Ernest Kiswaya Sumaili, François Bompeka Lepira, Fiston Ikwa Ndol Mbutiwi, Jean Robert Rissassy Makulo, Justine Busanga Bukabau, Patrick Parmba Izeidi, Jeannine Losa Luse, Stéphane Kalambay Mukendi, Désiré Kulimba Mashinda, Nazaire Mangani Nseka

**Affiliations:** 1 Division of Nephrology, University of Kinshasa, Kinshasa, Democratic Republic of the Congo; 2 Hemodialysis Unit of Ngaliema Medical Center, Kinshasa, Democratic Republic of the Congo; 3 Hemodialysis Unit of Provincial General Hospital of Kinshasa, Kinshasa, Democratic Republic of the Congo; 4 School of Public Health, University of Kinshasa, Kinshasa, Democratic Republic of the Congo; 5 Faculty of medicine, University of Kikwit, Kikwit, Democratic Republic of the Congo

## Abstract

**Background:**

Decreased residual urine volume (RUV) is associated with higher mortality in hemodialysis (HD). However, few studies have examined RUV in patients on HD in Sub-Saharan Africa. The aim of this study was to identify predictors of RUV among incident hemodialysis patients in Kinshasa.

**Methods:**

This historical cohort study enrolled 250 patients with ESRD undergoing hemodialysis between January 2007 and July 2013 in two hemodialysis centers in Kinshasa. RUV were collected over 24 h at the initiation of HD and 6 and 12 months later during the interdialytic period. We compared the baseline characteristics of the patients according to their initial RUV (≤ 500 ml/day vs >  500 ml/day) using Student’s t, Mann-Whitney U and Chi2 tests. Linear mixed-effects models were used to search for predictors of decreased RUV by adding potentially predictive baseline covariates of the evolution of RUV to the effect of time: age, sex, diabetes mellitus, hypertension, diastolic blood pressure, diuretics, angiotensin conversion enzyme inhibitors (ACEI), angiotensin receptor blockers, hypovolemia, chronic tubulointerstitial nephropathy, left ventricular hypertrophy and initial hemodialysis characteristic. A value of *p* < 0.05 was considered the threshold of statistical significance.

**Results:**

The majority of hemodialysis patients were male (68.8%, sex ratio 2.2), with a mean age of 52.5 ± 12.3 years. The population’s RUV decreased with time, but with a slight deceleration. The mean RUV values were 680 ± 537 ml/day, 558 ± 442 ml/day and 499 ± 475 ml/day, respectively, at the initiation of HD and at 6 and 12 months later. The use of ACEI at the initiation of HD (beta coefficient 219.5, *p* < 0.001) and the presence of chronic tubulointerstitial nephropathy (beta coefficient 291.8, *p* = 0.007) were significantly associated with RUV preservation over time. In contrast, the presence of left ventricular hypertrophy at the initiation of HD was significantly associated with decreased RUV over time (beta coefficient − 133.9, *p* = 0.029).

**Conclusions:**

Among incident hemodialysis patients, the use of ACEI, the presence of chronic tubulointerstitial nephropathy and reduced left ventricular hypertrophy are associated with greater RUV preservation in the first year of dialysis.

## Background

In Kinshasa, the capital City of the Democratic Republic of the Congo, the overall prevalence of chronic kidney disease (CKD) in the general population is estimated to be 12.4%, with 0.2% of individuals with end-stage renal disease (ESRD) [1]. Late referral is the underlying cause for the admission of most patients with ESRD. For these patients, renal replacement therapy, including hemodialysis (HD), guarantees better survival and good quality of life. Among the criteria for effective HD, the preservation of residual kidney function (RKF) or residual urine volume (RUV) plays an important role in contributing to the survival and quality of life of hemodialysis patients [2–4]. Patient demographic characteristics, comorbid disease and characteristics of dialysis treatment have been associated with a faster decline in RUV in dialysis patients. Among these factors, we identified increasing age [4, 5], female sex [4–7], diabetes [4, 5, 8], hypertension [6, 8, 9], left ventricular hypertrophy (LVH) [10], congestive heart failure [4, 5], proteinuria [11], frequent dialysis [7, 12–14], intradialytic hypotension [4, 5, 7, 11] and biocompatible membrane [4, 15] as relevant. In this context, the National Kidney Foundation’s Kidney Disease Outcome Quality Initiative (KDOQI) guidelines recommended the implementation of RKF preservation strategies in dialysis patients using RUV as a surrogate indicator [16, 17]. These strategies include the use of angiotensin-converting enzyme inhibitors (ACE) [18], angiotensin receptor blockers (ARB) [19] and diuretics [20]; the control of hypovolemia [21], obesity [22] and high blood pressure; the avoidance of nonsteroidal anti-inflammatory drugs, aminoglycosides and radiographic contrast agents [23]; the reduction of LVH [10]; a lower dose of dialysis [13]; the use of biocompatible dialysates and dialyzers and bicarbonate rather than acetate; and the early initiation of HD [5, 24, 25]. It is now accepted that the use of ultrapure water in HD helps preserve RKF [26]. Although the definition of the RKF varies across studies, RUV may emerge as a pragmatic alternative to calculate RKF [27]. Therefore, the search for factors that may contribute to the preservation of RUV in resource-limited settings should be a priority to improve the practice of HD. In sub-Saharan Africa, including the Democratic Republic of the Congo (DRC), there are no studies examining the predictors of RUV in patients undergoing HD. The present study assessed factors that may contribute to the preservation of residual urine volume in HD patients in Kinshasa.

## Methods

This historical cohort study enrolled 250 patients with ESRD undergoing HD in two HD centers in Kinshasa (Ngaliema Medical Center and Afia Medical Center) between January 2007 and July 2013. These facilities own Fresenius 4008B, 4008 s and, since 2010, 5008 s machines and a water treatment unit with double-pass reverse osmosis, which ensures the production of ultrapure water. Patients were alternately treated with hemodialysis (HD) or hemodiafiltration (HDF). The dialyzers used were made of polysulfone or high-flux membranes such as Helixone® Plus High-flux.

All patients with ESRD admitted for HD who received at least 4 weeks of renal replacement therapy were included in this study. Socio-demographic and anthropometric parameters of interest were age, gender, weight and height. For clinical parameters, systolic blood pressure (SBP) and diastolic blood pressure (DBP) were measured before the first session of HD using an electronic sphygmomanometer. Pulse pressure (PP) was calculated as the difference between SBP and DBP. The current medical treatment and complications at the initiation of HD were recorded on the appropriate form. Twenty-four-hour urine collection was used to measure RUV before beginning HD, and a similar urine collection protocol was used on days between dialysis 6 and 12 months later. The definition of volemia combined measurements of venous pressure, blood pressure and weight gain. For the evaluation of central venous pressure, we first performed a clinical assessment by placing the patient lying at 45° from the bed plane and appreciating the visibility of the jugular vein for a clinical estimation of 5–7 cm H2O. Patients with a value greater than 7 cm H2O benefited from the measure of central venous pressure. Hypervolemia was defined as a central venous pressure ≥ 13 cm H2O [28]. If the clinical estimate was below 5 cm H2O with decreased weight and blood pressure, patients were considered hypovolemic. Left ventricular hypertrophy (LVH) was noted when the thickness of the interventricular septum and posterior left ventricular wall was greater than 11 mm at the end of diastole or when the left ventricular mass was greater than or equal to 120 g/m2 [29]. Heart failure was defined on the basis of signs of pulmonary and peripheral stasis and a systolic ejection fraction of the left ventricle < 40% [30]. Chronic tubulointerstitial nephropathy was defined as End stage renal disease with the absence or modest degree of the two principal hallmarks of glomerular and vascular diseases of the kidney: salt retention, manifested by edema and hypertension; and proteinuria, which usually is modest and less than 1 to 2 g/d in TIN.

### Statistical analysis

The continuous variables are presented as an average with the standard deviation or median values and interquartile range (IQR), and the categorical variables are presented in the form of absolute frequency (percentage).

We compared the baseline characteristics of the patients according to their initial RUVs (≤ 500 ml/day vs >  500 ml/day) using Student’s t, Mann-Whitney U and Chi2 tests, as appropriated.

We described the average populational and individual evolution of RUV repeated-measurements by graphical analysis. The RUV medians (IQR) at each assessment time are presented based on baseline categorical covariates. For the continuous covariates, the Pearson correlation coefficient r was presented.

For modelization, we first chose a linear mixed-effects model to analyze the effect of time alone on the decline in RUV (model 1). As the course of RUV mainly showed a non-linear pattern in the repeated-measurements analyses, this model allows a random intercept, a random slope and the quadratic term of time in fixed effects, with an unstructured variance-covariance matrix. Next, we constructed model 2 based on model 1 by adding baseline covariates as potential predictors of the evolution of RUV: age (years), sex (female vs male), diabetes mellitus (yes vs no), hypertension (yes vs no), diastolic blood pressure (mm Hg), the use of diuretics (yes vs no), ACE inhibitors (yes vs no) and angiotensin receptor blockers (ARB, yes vs no), hypovolemia (yes vs no), Chronic tubulointerstitial nephropathy (yes vs no), left ventricular hypertrophy (yes vs no) and initial hemodialysis characteristic (≥ 3 vs ≤ 2 times weekly). Congestive heart failure was not chosen because of its correlation with left ventricular hypertrophy. Proteinuria was not tested because of the importance of missing data. In addition, using model 2 (main model), we tested other models, each time adding a term of time interactions with each of the baseline covariates tested in model 2, with the exception of age. For each of these models, only the variables that were significantly associated with the evolution of RUV were included in the final models. We conducted the residuals analysis to ensure the validity of the model assumptions, and plotted the predicted marginal means of RUV over time. Finally, we performed a sensitivity analysis based on the final model 2 by fitting a model allowing the quadratic term of time as a random effect (individuals curves) and those with the exchangeable and autoregressive residual AR(1) covariance structures instead of the unstructured covariance matrix. We used the Akaike information criterion (AIC) to compare these models.

SPSS software version 24 and Stata/IC version 14.2 (StataCorp LP, College Station, Texas, USA) were used to carry out all statistical analyses. A value of *p* < 0.05 was considered the threshold of statistical significance.

The Ethics Committee of the Faculty of Medicine, University of Kinshasa approved the implementation of this study.

## Results

### Baseline characteristics of the patients

The majority of dialysis patients were male (68.8%, sex ratio 2.2), with a mean age of 52.5 ± 12.3 years (Table 1). The initiation of HD for the entire group corresponded to a median (IQR) estimated glomerular filtration rate (eGFR) according to MDRD, serum creatinine and plasma urea of 5.0 (3.0–8.0) ml/min/1.73 m^2^, 12.0 (8.2–17.8) mg/dl and 197 (147–288) mg/dl, respectively. The median values (IQR) of eGFR, serum creatinine and plasma urea of HD patients with and without preserved IUV (initial urine volume) were 6.0 (4.0–8.0) vs 4.0 (3.0–8.0) ml/min/1.73 m^2^ (*p* = 0.023), 11.0 (8.0–16.0) vs 13.0 (8.4–20.0) mg/dl (*p* = 0.036) and 187 (139–261) vs 223 (168–331) mg/dl (*p* = 0.004), respectively.

**Table 1:** Clinical and biological characteristics of the patients at the initiation of hemodialysis

Variables	All group *n* = 250	Initial urine volume (ml/day)		*p*
		≤ 500 *n* = 123	> 500 *n* = 127	
Age, years	52.5 ± 12.4	52.1 ± 13.3	52.8 ± 11.5	0.683
Sex, female, n (%)	78 (31.2)	44 (35.8)	34 (26.8)	0.125
Diabetes mellitus, n (%)	98 (39.2)	48 (39.0)	50 (39.4)	0.955
SBP, mm Hg	153.8 ± 27.2	153.4 ± 27.3	154.1 ± 27.3	0.821
DBP, mm Hg	84.7 ± 18.3	84.0 ± 17.5	85.2 ± 19.1	0.604
PP, mm Hg	69.1 ± 20.8	69.4 ± 19.9	68.9 ± 21.8	0.861
Hypertension, n (%)	217 (86.8)	104 (84.6)	113 (89.0)	0.302
Diuretics, n (%)	133 (53.2)	57 (46.3)	76 (59.8)	0.032
ACE inhibitors, n (%)	138 (55.2)	55 (44.7)	83 (65.4)	0.001
ARB, n (%)	29 (11.6)	12 (9.8)	17 (13.4)	0.370
Hypovolemia, n (%)	40 (16.0)	12 (9.8)	28 (22.1)	0.008
Hypervolemia, n (%)	95 (38.0)	68 (55.3)	27 (21.3)	< 0.001
eGFR-MDRD, ml/min/1.73 m^2^	5.0 (3.0–8.0) *(n = 240)*	4.0 (3.0–8.0) *(n = 115)*	6.0 (4.0–8.0) *(n = 125)*	0.023
Serum creatinine, mg/dl	12.0 (8.2–17.8) *(n = 240)*	13.0 (8.4–20.0) *(n = 115)*	11.0 (8.0–16.0) *(n = 125)*	0.036
Serum urea, mg/dl	197 (147–288) *(n = 239)*	223 (168–331) *(n = 115)*	187 (139–261) *(n = 124)*	0.004
Serum potassium, mmol/l	5.2 ± 1.4 *(n = 239)*	5.4 ± 1.4 *(n = 115)*	4.9 ± 1.3 *(n = 124)*	0.007
Hemoglobin level, g/dl	8.5 ± 2.2 *(n = 249)*	8.2 ± 2.2 *(n = 122)*	8.8 ± 2.2 *(n = 127)*	0.042
Proteinuria, g/24 h	*(n = 77)*	*(n = 26)*	*(n = 51)*	0.343
< 1, n (%)	22 (28.6)	6 (23.1)	16 (31.4)	
1–3, n (%)	41 (53.2)	13 (50.0)	28 (54.9)	
> 3, n (%)	14 (18.2)	7 (26.9)	7 (13.7)	
Serum albumin, g/l	36.8 ± 7.3 *(n = 234)*	35.6 ± 7.4 *(n = 116)*	38.1 ± 7.0 *(n = 118)*	0.007
Interstitial initial nephropathy, n (%)	23 (9.2)	9 (7.3)	14 (11.0)	0.311
Left ventricular hypertrophy, n (%)	101 (42.8) *(n = 236)*	55 (47.8) *(n = 115)*	46 (38.0) *(n = 121)*	0.200
Congestive heart failure, n (%)	86 (34.4)	47 (38.2)	39 (30.7)	0.212
≥ 3 times hemodialysis weekly, n (%)	116 (46.4)	60 (48.8)	56 (44.1)	0.458

Data are expressed as the mean ± standard deviation, median (interquartile range) or absolute frequency (relative frequency in percent)

*Abbreviations*: *SBP* systolic blood pressure, *DBP* diastolic blood pressure, *ACE* angiotensin conversion enzyme, *ARB* angiotensin receptor blockers, *eGFR* estimated glomerular filtration ratio, *MDRD* modification of diet in renal disease

Patients with preserved initial RUV, compared to those with an initial RUV of less than 500 ml/day, were often on diuretics (60% vs 46%, respectively; *p* = 0.032) and ACE inhibitors (65.4% vs 44.7%, respectively; *p* = 0.001). They had less hypervolemia (21.3% vs 55.3%, respectively; *p* < 0.001), hyperkalemia (4.9 ± 1.3 vs 5.4 ± 1.4 mEq/l, respectively; *p* = 0.007) and anemia (Hb 8.8 ± 2.2 vs 8.2 ± 2.2 g/dl, respectively; *p* = 0.042). In addition, their serum albumin level was higher (38.1 ± 7.0 vs 35.6 ± 7.4 g/l, respectively; *p* = 0.007).

### Evolution of urine volume in patients undergoing hemodialysis

In total, 454 repeat measurements of RUV were performed in patients at three evaluation stages: 250 measurements (100% of patients) at the initiation of HD, 127 (50.8%) at 6 months and 77 (30.8%) at 12 months from the start of HD. The RUV varied between 0 and 2740 ml/day, with a median value (IQR) of 500 ml/day (200–1000 ml/day).

**Fig. 1:**
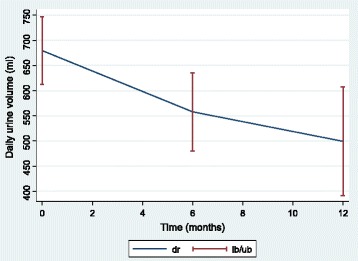
Population means (95% confidence interval bars) of residual urine volume at each assessment time

**Fig. 2:**
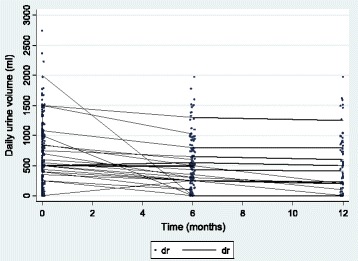
Scatterplot of residual urine volumes over time, individual trajectories for 50 randomly chosen patients

Figure 1 shows that the population RUV decreased with time but with a slight deceleration. This trend is also observed at the individual level (Fig. 2). The mean RUV values were 680 ± 537 ml/day, 558 ± 442 ml/day and 499 ± 475 ml/day, respectively, at the initiation of HD and 6 and 12 months later.

### Urine volumes of patients according to their baseline characteristics

Urine volume (median) decreased over time in all patients, except in non-hypertensive patients, whose urine volume tended to increase over time (Table 2). Roughly, at each assessment time, the median urine volume was higher in men than in women, in patients with hypovolemia, in those with chronic tubulointerstitial nephropathy, and in those treated with diuretics, ACE inhibitors and ARB compared to the others. On the other hand, urine volume was lower in diabetics and hypertensive patients (as of the 2nd assessment), patients with left ventricular hypertrophy or heart failure, and those with initially ≥3 sessions of HD per week compared to the others. Urine volume was not correlated with age (Pearson *r* = 0.004) or diastolic blood pressure (Pearson’s r value = − 0.010).

**Table 2:** Evolution of urine volume (ml/day) in patients undergoing hemodialysis according to baseline categorical characteristics

	At initiation		At 6th month		At 12th month	
	n	Median (IQR)	n	Median (IQR)	n	Median (IQR)
All patients	250	550 (200–1000)	127	500 (250–830)	77	400 (100–840)
Sex						
Male	172	600 (235–1008)	93	500 (250–800)	53	410 (158–773)
Female	78	500 (200–1050)	34	450 (95–1000)	24	300 (26–938)
Diabetes mellitus						
No	152	535 (250–1050)	75	500 (250–900)	46	500 (200–850)
Yes	98	550 (190–1000)	52	475 (231–720)	31	200 (0–750)
Hypertension						
No	33	500 (90–968)	10	770 (0–950)	5	840 (325–925)
Yes	217	600 (250–1050)	117	500 (250–800)	72	325 (100–784)
Diuretics						
No	117	500 (135–1000)	58	375 (100–1000)	36	325 (0–888)
Yes	133	730 (325–1035)	69	500 (300–795)	41	450 (200–773)
ACE inhibitors						
No	112	400 (100–1000)	44	350 (100–875)	27	275 (0–795)
Yes	138	750 (400–1051)	83	500 (300–830)	50	500 (200–863)
ARB						
No	221	500 (200–1000)	112	500 (250–800)	65	400 (100–795)
Yes	29	800 (300–1060)	15	650 (350–900)	12	455 (250–900)
Hypovolemia						
No	210	500 (200–1013)	115	500 (225–800)	71	300 (50–850)
Yes	40	800 (300–1038)	12	710 (425–963)	6	575 (475–868)
Interstitial initial nephropathy						
No	227	500 (200–1000)	119	500 (250–800)	72	325 (100–773)
Yes	23	975 (280–1490)	8	900 (750–1250)	5	850 (700–950)
Left ventricular hypertrophy						
No	135	730 (250–1100)	70	500 (250–900)	45	400 (200–875)
Yes	101	500 (200–1000)	49	450 (100–840)	31	350 (0–750)
Congestive heart failure						
No	164	610 (200–1054)	78	500 (275–863)	50	375 (179–913)
Yes	86	500 (238–1000)	49	480 (100–790)	27	410 (50–650)
Initial hemodialysis characteristic						
≤ 2 times weekly	134	600 (200–1000)	62	500 (300–863)	30	455 (100–843)
≥ 3 times weekly	116	500 (200–1043)	65	450 (113–800)	47	300 (50–850)

*Abbreviations*: *IQR* interquartile range, *ACE* angiotensin conversion enzyme, *ARB* angiotensin receptor blockers

### Factors significantly associated with the preservation/decrease of urine volume (ml/day) over time

As the Table 3 shows, time has a significant quadratic effect on RUV. The negative beta coefficient (for time) shows that the average RUV decreased with time. The positive coefficient (for the quadratic term, time^2^) shows that there was a deceleration of this decline (the decline slowed down with time). The use of ACE inhibitors at the initiation of HD (beta coefficient of 219.5, *p* < 0.001) and the presence of interstitial initial nephropathy (beta coefficient of 291.8, *p* = 0.007) were the baseline characteristics significantly associated with the preservation of RUV over time. In contrast, the presence of left ventricular hypertrophy at the initiation of HD was significantly associated with decreased RUV over time (beta coefficient of − 133.9, *p* = 0.029). There were no significant interactions between the time variable and the baseline characteristics of patients.

**Table 3:** Factors significantly associated with the preservation/decrease of residual urine volume (ml/day) over time

	Model 1			Model 2			Model 3			Model 4		
	β	SE	*p*	β	SE	*p*	β	SE	*p*	β	SE	*p*
Intercept	679.6	33.5	< 0.001	595.7	55.9	< 0.001	595.1	55.3	< 0.001	595.6	55.6	< 0.001
Time (month)	−36.6	6.5	< 0.001	−37.5	6.8	< 0.001	−40.5	10.1	< 0.001	−40.8	8.8	< 0.001
Time*time (month^2^)	1.4	0.5	0.002	1.4	0.5	0.002	1.7	0.9	0.047	1.7	0.7	0.019
ACE inhibitors, yes vs no				219.5	62.1	< 0.001	237.5	62.5	< 0.001	236.2	62.7	< 0.001
Left ventricular hypertrophy, yes vs no				−133.9	61.2	0.029	− 148.4	61.8	0.016	−149.1	62.1	0.016
Interstitial initial nephropathy, yes vs no				291.8	108.9	0.007	256.8	108.1	0.018	262.4	108.6	0.016

Model 1: Fixed effects: intercept, time, time^2^; Random effects: intercept, time; covariance structured: unstructured; AIC: 6756.8. Model 2 (main model): model 1 + baseline covariates (age, sex, diabetes mellitus, hypertension, diastolic blood pressure, diuretics, ACE inhibitors, angiotensin receptor blockers, hypovolemia, interstitial initial nephropathy, left ventricular hypertrophy and initial hemodialysis characteristic) as fixed effects; AIC: 6410.8. Model 3: model 2 with exchangeable covariance structure; AIC: 6427.3. Model 4: model 2 with autoregressive residual (1) covariance structure; AIC: 6420.4

*Abbreviation*: *ACE* angiotensin conversion enzyme, *SE* standard error, *AIC* Akaike information criterion

For the sensitivity analysis, the model in which the quadratic term of time was allowed as a random effect failed to converge. However, the model 2 containing an unstructured covariance matrix (AIC = 6410.8.) provided a better fit than those with the exchangeable (AIC = 6427.3) and the AR(1) (AIC = 6420.4) covariance structures.

**Fig. 3:**
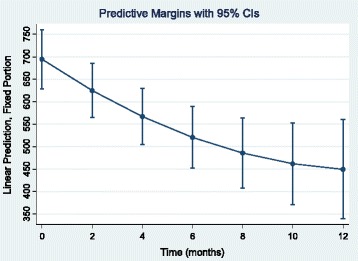
Model 2 predicted marginal means of residual urine volumes (ml) over time with 95% confidence intervals (CIs)

Figure 3 shows the marginal average of VUR predicted by the model 2 and better displays the quadratic pattern of RUV over time.

## Discussion

This study assessed predictors of RUV preservation in HD patients. The mean RUV at the initiation of HD was as high as 680 ml. This is significantly elevated compared to the data reported in the CHOICE study [31]. RUV was also better preserved in the present study than in the CHOICE study.

Indeed, one year after the initiation of HD, 60% of patients in this study still yielded > 250 ml/day as opposed to 23.2% in the CHOICE study [31]. Numerous comorbidities and the more advanced ages of HD patients in Western countries could explain this difference.

When we analyze the effect of time alone, we note that the speed of the decline in RUV slowed down with time. This effect remained present even after adjustments for ACE inhibitors use, chronic tubulointerstitial nephropathy and LVH in model 2. This slowing of the decline in urine volume with time could be attributed to an improvement in the technique of dialysis with the introduction of the HDF technique in the two study centers since 2010. HDF is characterized by a small decrease in RUV, similar to peritoneal dialysis.

The present study did not show an association between urine volume and age or sex. These results are contrary to those of the literature [4–7]. However, Nechita et al., in a study of a cohort of 216 patients with stage 5 chronic kidney disease (CKD) beginning chronic HD, also failed to find a significant association between residual diuresis and age or sex [32]. It is possible that the small sample size did not reveal the influence of age or sex on the loss of urine volume. Another reason may be related to the fact that urine volume is the result of glomerular filtration, tubular reabsorption and the influence of diuretic therapy. In addition, in CKD, the concentration and dilution tubular capacity is altered as a result of tubulointerstitial fibrosis and increased tubular resistance by ADH [33, 34].

In the present study, independent predictors of urine volume were the use of ACE inhibitors, chronic tubulointerstitial nephropathy and LVH.

The beneficial role of ACE inhibitors on RKF and RUV in HD has been demonstrated in the analysis of the U.S. Renal Data System data, a study of 2211 incident dialysis patients with RKF defined by UV > 200 ml/day. This study found that the use of an ACE inhibitor was independently associated with a decreased risk of UV loss [5]. Several other studies have shown the benefit of ACE inhibitors on RKF and RUV in HD patients [35–38]. The prospective study by Xydakis et al. proved that ACE inhibitors have a significant effect on preserving RKF in patients starting dialysis, at least during the first 12 months from the initiation of the HD, with RKF defined as residual GFR and RUV [18]. This beneficial effect of ACE inhibitors can be explained not only by the control of blood pressure parameters and by the anti-proteinuric effect but also by their action against inflammation, malnutrition and glomerular and interstitial fibrosis linked to the deleterious action of angiotensin II [38]. In addition, blood contact with the HD filter membrane causes a cascade of reactions, including activation of mononucleated cells that are responsible for several inflammatory mediators, such as IL-1_β_, IL-6, TNFα, reactive oxygen species (ROS), and nitrogen monoxide, and the release of proteins from the acute phase of inflammation, such as C reactive protein [38]. The use of ACE inhibitors decreases serum TNF alpha and C reactive protein levels [39], resulting in a reduction in inflammation and oxidative stress and subsequently better preservation of RKF and RUV. Kidney Disease Outcomes Quality Initiative guidelines currently recommend the use of ACE inhibitors in the preservation of RKF and in the control blood pressure in patients with RUVs in excess of 100 ml [16].

The principal manifestations of TIN are those of tubular dysfunction. The tubulointerstitial lesions are localized either to the cortex or medulla. The extent of damage determines the severity of tubular dysfunction. Disruption of these structures, therefore, results in different degrees of nephrogenic diabetes insipidus and clinically manifests as polyuria and nocturia [33, 34]. A close correlation exists between the severity of chronic tubulointerstitial nephropathy and impaired renal tubular and glomerular function. Chronic tubulointerstitial nephropathy usually preserve the RUV more often in this study. Tubular dysfunction probably justified the preservation of RUV in patients with chronic tubulointerstitial nephropathy [34].

LVH is an important predictor of cardiovascular mortality and morbidity in dialysis patients. Hypertension, diabetes, increased body mass index, gender, age, anemia, and hyperparathyroidism have been described as risk factors for LVH in HD patients [40]. LVH is also recognized in the literature as a predictor of the loss of RUV [4, 5, 10]. LVH was negatively correlated with RUV [40]. The results of our study corroborate the information described in the literature regarding the negative effect of LVH on RUV. The mechanisms by which LVH favors altered RUV are both hemodynamic and neurohormonal. Diastolic and/or systolic dysfunction decrease renal perfusion pressure and effective ultrafiltration pressure [10] and increase renal venous pressure and water and sodium retention, contributing substantially to the decline in RUV. In addition, sympathetic stimulation, renin-angiotensin-aldosterone system activation, inflammation, oxidative stress and endothelial dysfunction also contribute to alterations of RUV by renal fibrosis [41]. In CKD, a veritable vicious circle is established between LVH and RUV. The loss of RUV favors hypervolemia and elevated blood pressure, two factors that favor LVH, which will exacerbate the loss of RUV by hypoperfusion. To combat LVH, the use of ACE inhibitors is the treatment of choice. A comparison between ACE inhibitors and other drug controls showed that ACE inhibitors cause a greater reduction in LVH in HD patients [42]. Another strategy is to increase the duration and frequency of HD sessions [43]. If this strategy has the capacity to reduce RUV in conventional HD [13, 14], this is not the case in hemodiafiltration (HDF). High-efficiency post-dilution on-line HDF reduces LVH without altering RUV [44].

Given the influence of the time spent in HD on RUV, it is necessary to make efforts to improve the practice of HD. The quality of the water treatment (ultrapure water) [26], the use of biocompatible dialysates and dialyzers [24, 25], and the prevention of peridialytic hypotension [5, 7, 11] must be taken into account.

The interpretation of the results of the present study should consider some limitations. First, the retrospective characteristic of the study design precludes the establishment of an all cause-effect relationship. Second, the small sample size did not confer much power to the statistical tests to identify additional associations. Without urine creatinine available, the accuracy of 24-h urine output in the present study could not be certified. However, despite these limitations, the present study is the first to evaluate independent predictors of RUV in Sub-Saharan Africa.

## Conclusion

In the present study, the use of ACE inhibitors, chronic tubulointerstitial nephropathy and LVH reduction emerged as the main independent predictors of RUV preservation over time. The use of ACE inhibitors and HDF should be preferred for the optimal management of HD patients.

## Abbreviations

ACE: Angiotensin conversion enzyme
CKD: Chronic Kidney Disease
DBP: Diastolic blood pressure
eGFR: glomerular filtration rate
ESRD: End-stage chronic renal disease
HD: Hemodialysis
HDF: Hemodiafiltration
LVH: Left ventricular hypertrophy
MDRD: Modification of Diet in Renal Disease
P: Statistical significance
PP: Pulse pressure
RKF: Residual kidney function
RUV: Residual urine volume
SBP: Systolic blood pressure
